# Incidence of lymphomas in inflammatory bowel disease: report of an emblematic case, systematic review, and meta-analysis

**DOI:** 10.3389/fmed.2023.1172634

**Published:** 2023-05-03

**Authors:** Maria Francesca Russo, Annalisa Diddoro, Alessandra Iodice, Carola Severi, Lidia Castagneto-Gissey, Giovanni Casella

**Affiliations:** ^1^Department of Surgery, Sapienza University of Rome, Rome, Italy; ^2^Department of Internal Medicine and Medical Specialties, Sapienza University of Rome, Rome, Italy

**Keywords:** lymphoma, inflammatory bowel disease, ulcerative colitis, proctocolectomy, biologic agents

## Abstract

**Introduction:**

Over the past 20 years, the increasing use of combined therapy with immunosuppressants and biologic agents has markedly reduced the use of steroids in the management of inflammatory bowel diseases (IBD). However, medical therapy seems to promote, in the long run, carcinogenesis resulting in an increased risk of developing different types of malignancies, including lymphomas. The aim of this study was to systematically review the current incidence and prognosis of lymphoid neoplasms occurring in patients with IBD.

**Methods:**

Studies analyzing the incidence of lymphomas in subjects of age >18 years affected by IBD were included in this systematic review and meta-analysis. Studies focusing on pediatric populations, not reporting person-years of follow-up, or with a duration < 1 year were excluded. PubMed, Embase, Web of Science Core Collection, and Cochrane Central Register were searched from inception through January 2022. Publication bias within studies was assessed using Begg's and Egger's tests and random effects model. Quantitative results were synthesized using relative-risk meta-analysis. PRISMA guidelines were used to carry out this systematic review (PROSPERO Registration Number: CRD42023398348).

**Results:**

A total of 345 studies published between 1985 and 2022, with a total of 6,17,386 patients were included in the meta-analysis. Substantial heterogeneity between studies prevented the pooling of estimates (*I*^2^ = 97.19%). Evidence of publication bias was overall low (*p* = 0.1941). Patients affected by Crohn's disease (CD) were 1,86,074 (30.13%), while 2,78,876 (46.17%) were diagnosed with UC. The remaining 23.7% of cases were diagnosed with indeterminate colitis. Immunomodulators and biologic therapy were used in 24,520 (5.27%), and 17,972 (3.86%) patients, respectively. Reported incidence rates for lymphoma in IBD ranged from 0.0/100,000 person/years (py) (95% CI 0.0–3.7/100,000) to 89/100,000 py (95% CI 36–160/100,000). Reported incidence rates of lymphoma in CD ranged from 0.0/100,000 py (95% CI 0.0–3.7/100,000) to 91/100,000 py (95% CI 18–164/100,000). For UC, the incidence rate ranged from 0.0/100,000 py (95% CI 0.0–3.7/100,000) to 95/100,000 py (95% CI 0–226/100,000). Male-to-female ratio was ~4:1. Therapy with immunomodulators was directly associated with an increased incidence of lymphoma (*p* < 0.0001). Evidence of publication bias was overall low (*p* = 0 .1941).

**Conclusions:**

The evidence arising from this study highlights a correlation between the use of immunomodulators and subsequent lymphoma development. Combined multidisciplinary approach and long-term follow-up are warranted in order to decrease mortality deriving from the coexistence of both conditions.

**Systematic review registration:**

Identifier: CRD42023398348.

## 1. Introduction

Inflammatory bowel diseases (IBD), namely Crohn's disease (CD) and ulcerative colitis (UC), are chronic, idiopathic, lifelong inflammatory conditions of the gastrointestinal tract (1). The foundation of treatment in IBD is represented by medical therapies that diminish the mucosal inflammatory response. During the past 20 years, an increasing use of combined therapy with immunosuppressants and biologic agents has markedly reduced the use of steroids (2). However, medical therapy seems to promote, in the long run, carcinogenesis resulting in an increased risk of developing different types of malignancies, including lymphomas (3). Specifically, thiopurines are associated with an increased risk of lymphoma development (4–6). For what concerns anti-TNF drugs, it is unclear whether the greater risk of lymphoma is due to biologic therapy itself, or whether the augmented risk is explained by past or concomitant exposure to immunosuppressants, namely thiopurines (7–10). Additionally, the risk of lymphoma in patients who are exposed to combination therapy is still unknown (5, 10). In the absence of immunosuppressive therapies or biologic agents the absolute risk of developing lymphomas in the IBD subpopulation is quite low and does not seem to exceed that of the general population (11). Furthermore, Epstein-Barr virus is significantly involved in lymphoma development in patients with IBD, accounting for at least 40% of cases when patients are treated with immunomodulators (12).

According to a French national cohort study of an estimated 1,89,289 patients affected by IBD, those receiving combination therapy of thiopurine and anti-TNF agents had a 6-fold greater risk of lymphoma compared to those receiving monotherapy of either drug (13). A 2018 meta-analysis revealed the presence of a strong correlation between lymphoma risk and anti-TNF exposure (14). On the contrary, a recent review of observational data was unable to identify or rule out any link between lymphomas and anti-TNF medication in IBD patients (15).

The aim of this study was to systematically review the current incidence and prognosis of lymphoid neoplasms occurring in patients with IBD, additionally describing an emblematic case of a 56-year-old female patient affected by UC and a subsequent delayed diagnosis of large bowel lymphoma.

## 2. Case report

A 56-year-old Jehovah's Witness female patient, previously diagnosed with UC in 2018, and under medical treatment with mesalazine, salazopirine and corticosteroids, was admitted to our Department in August 2022, due to recurrent low-grade fever, malaise, asthenia, anemia (Hb 7.1 g/dL) and bloody diarrhea. On physical examination, the patient presented with abdominal pain in the left upper quadrant. The patient had a previous history of Hashimoto's disease, rheumatoid arthritis, and immune-mediated pericarditis. Furthermore, the patient was treated in the past with biologic agents (infliximab) and immunosuppressants (azathioprine).

A colonoscopy was performed due to the suspicion of UC exacerbation, which showed edematous mucosa, erythema, hyperemia, granularity, loss of vascular markings and mucosal friability. Immunochemistry to cytomegalovirus was negative.

**Figure 1:**
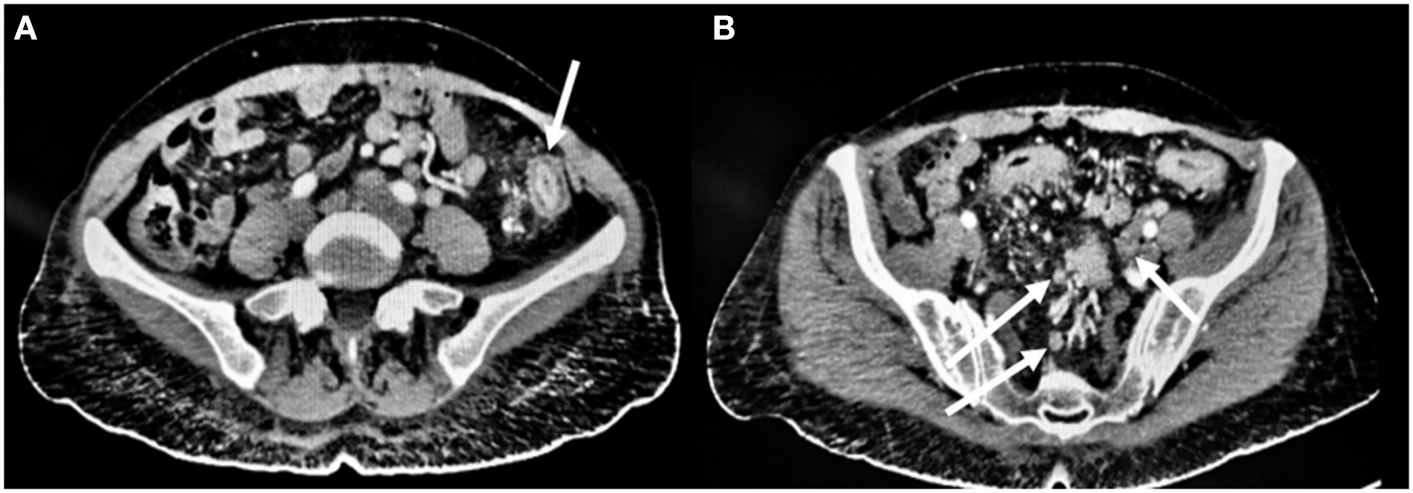
Pre-operative CT scan findings. **(A)** Circumferential, symmetrical wall thickening with fold enlargement at the level of the sigmoid colon (arrow) along with inhomogeneous attenuation and proliferation of perirectal fat. **(B)** Concomitant multiple reactive lymph nodes (arrow) in the mesentery, perirectal and parasigmoid tissue.

Additionally, an abdomen CT scan was performed, showing circumferential, symmetrical wall thickening with fold enlargement at the level of the sigmoid colon along with inhomogeneous attenuation and proliferation of perirectal fat. Concomitant multiple reactive lymph nodes in the mesentery, perirectal and parasigmoid areas were also present (Figure 1).

During hospital stay the patient was started on intravenous iron infusion and high-dose steroids, with improvement of symptoms and reduction of per rectal bleeding.

Due to the recurrence of the aforementioned symptoms which became refractory to medical therapy in September 2022, the patient underwent laparoscopic total proctocolectomy with end ileostomy.

On post-operative day 4 the patient presented with high-grade fever, which spontaneously resolved. On post-operative day 7, the patient was discharged home in good clinical conditions. The stoma was healthy and productive.

**Figure 2:**
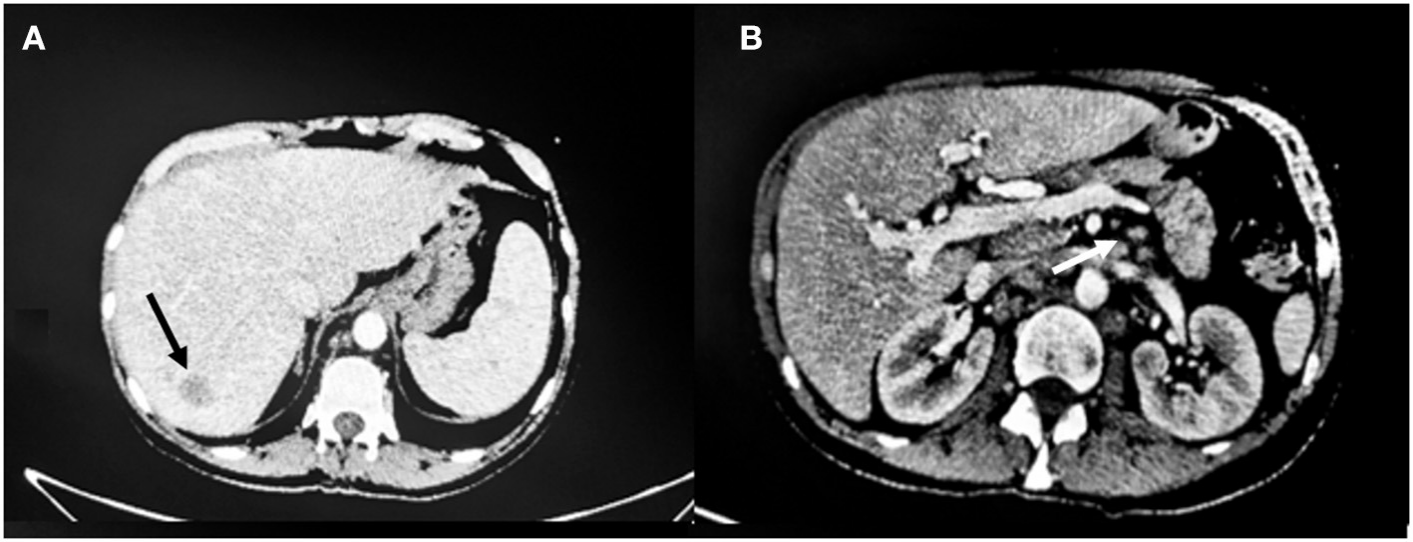
CT scan findings 20 days post-operatively. **(A)** The arrow shows one of the three hypointense hepatic lesions and periportal halo. **(B)** Dilatation of the intrahepatic biliary tree and inhomogeneous enhancement of the hepatic parenchyma as for cholangitis with increase in size and number of the splenic hilar (arrow), hepatic hilar and paraaortic lymph nodes.

Due to recurrent low-grade fever, on post-operative day 20, the patient underwent a CT scan which showed at least three hypointense hepatic lesions with periportal halo; dilatation of the intrahepatic biliary tree on the left and inhomogeneous enhancement of the hepatic parenchyma as for cholangitis. Rounded lesions with blurred margins were also present in the splenic parenchyma. The CT scan also showed an increase in size and number of the splenic hilar, hepatic hilar and retrocrural lymph nodes as well as lumbar-aortic, paraaortic and aorto-caval lymphomegaly. These findings were highly suspicious for a lymphoproliferative disease (Figure 2).

Histopathological analysis of the surgical specimen showed intestinal localization of EBV-related lymphoproliferative disease with histological and immunophenotypic features of classic Hodgkin lymphoma with mixed cellularity, arising from chronic inflammatory disease of the large bowel.

**Figure 3:**
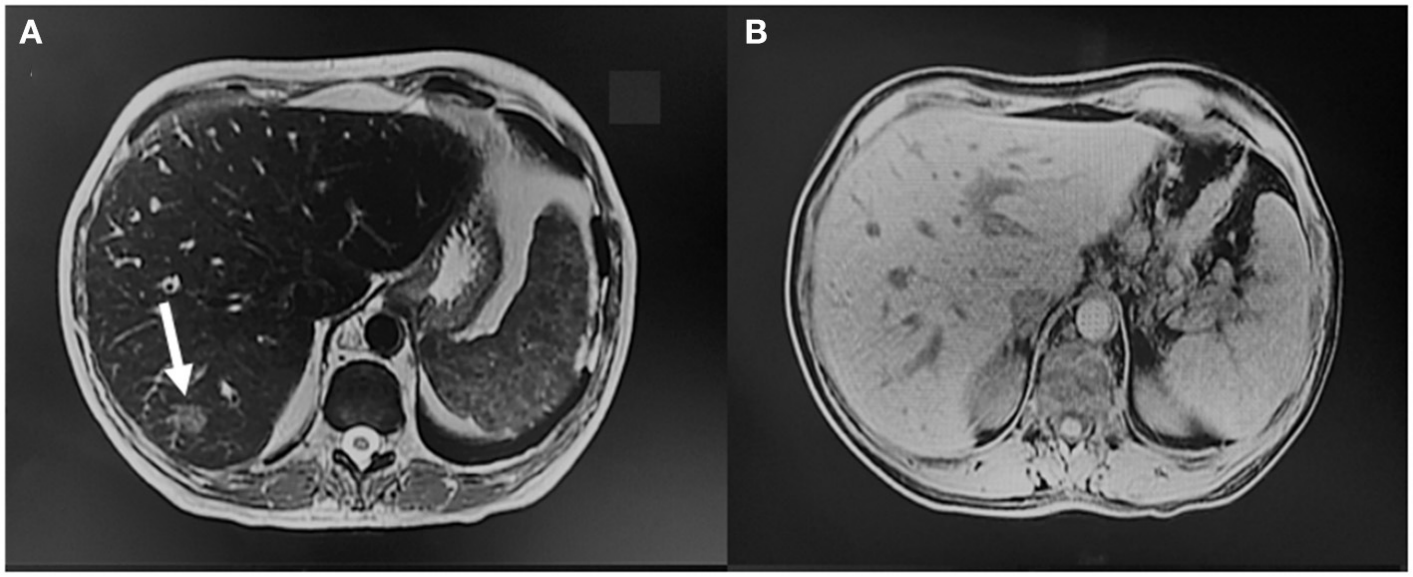
MRI findings during subsequent hospital admission. **(A, B)** Hepatomegaly and size reduction of the hepatic lesions previously described (arrow), with reduction of the multiple reactive abdominopelvic lymph nodes.

In October 2022 the patient was admitted to the Emergency Department due to high-grade fever and anemia and was later transferred to the Gastroenterology Unit where she underwent additional imaging. An MRI of the abdomen showed hepatomegaly and reduction in size of the hepatic lesions previously described. The multiple reactive abdominopelvic lymph nodes were also reduced (Figure 3).

**Figure 4:**
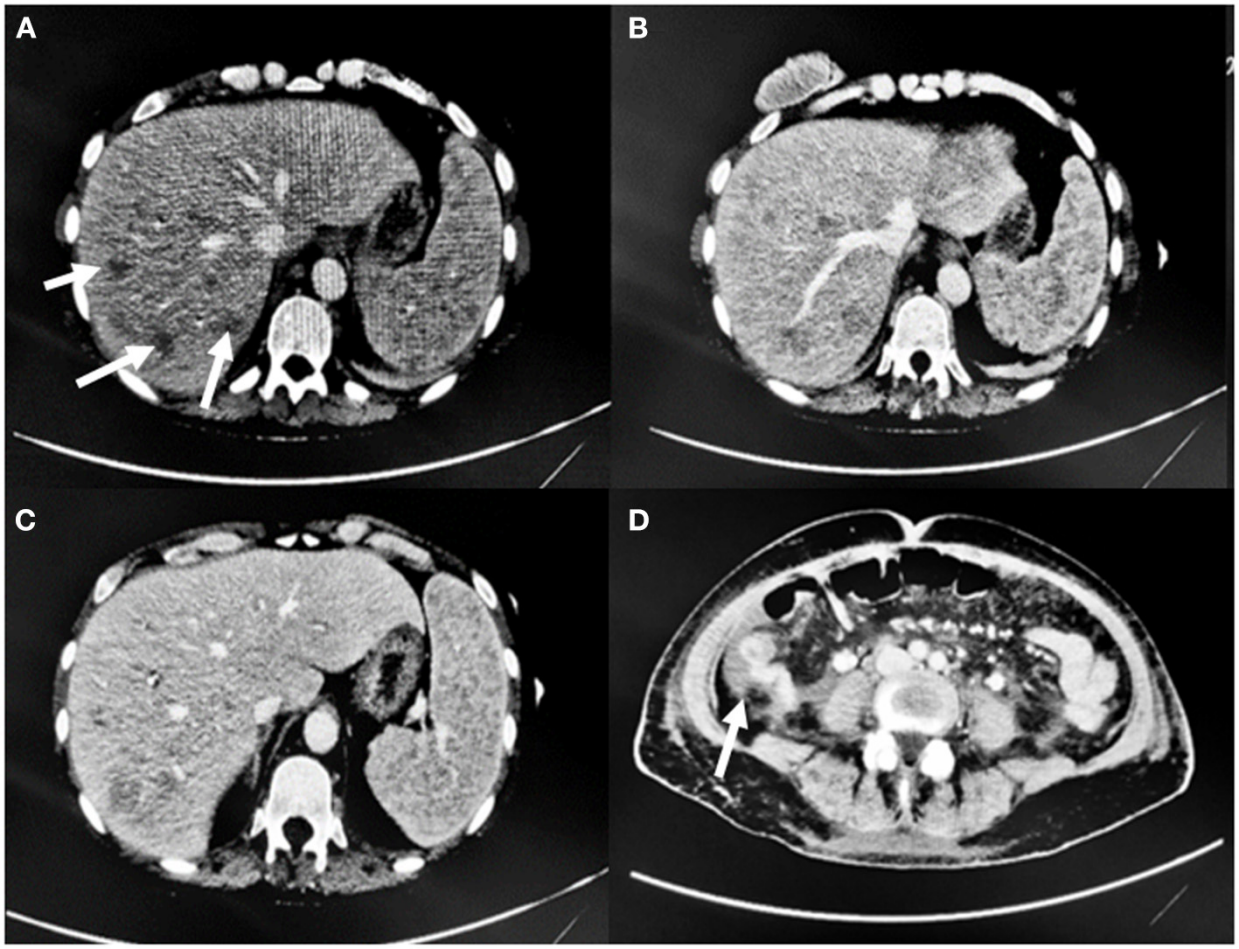
CT scan findings. **(A, B)** Increase in size and number of hepatic lesions (arrows), **(C)** Splenomegaly. **(D)** Fluid effusion in the right colic gutter.

Due to the worsening of her general conditions, she underwent an abdominal ultrasound which showed increased subhepatic and peripancreatic lymph nodes. A follow-up CT scan which showed an increase in the size of hepatic lesions, fluid collection around the gallbladder and common bile duct dilation. Furthermore, fluid effusion in the left colic gutter and in the pelvis was also present. All these findings were compatible with obstructive cholangitis. Splenomegaly and additional lymph nodes determining compressive phenomena on the inferior vena cava were also seen (Figure 4).

The patient died shortly after in November 2022 due to hepatic failure.

## 3. Methods

### 3.1. Search strategy and selection of studies

This study provides a systematic review and meta-analysis of previously published data, which was carried out according to the Preferred Reporting Items for Systematic reviews and Meta-Analyses (PRISMA) Statement. This study was registered to the PROSPERO International prospective register of systematic reviews (Registration Number: CRD42023398348).

The Population, Intervention, Comparison, Outcomes and Study (PICOS) framework was used to formulate the study protocol as follows. Population: all persons diagnosed with IBD eventually developing lymphoma. Papers that explored juvenile and pediatric cases only were excluded. Outcome: incidence rate of lymphoma in IBD population was the primary outcome measure. This review did not have an intervention or comparator group.

The search was performed using the following electronic databases without any year restriction, from inception through January 30, 2022: PubMed, Embase, the Web of Science Core Collection, and the Cochrane Central Register of Controlled Trials. All abstracts in the English language were screened for applicability. A manual search using the following keywords extracted from the Medical Subjects Heading (MeSH) was made: “inflammatory bowel disease” AND “IBD” AND “Crohn's disease” AND “ulcerative colitis” AND “lymphoma” AND “incidence” AND “prevalence” AND “malignancies”.

### 3.2. Eligibility criteria

All studies evaluating persons of age >18 years and diagnosed with IBD eventually developing lymphoma were included in the review. Studies were included if they reported incidence or provided sufficient information to accurately calculate the incidence of lymphomas in adult IBD patients. Studies focusing on pediatric populations, not reporting person-years of follow-up, or with a duration <1 year were excluded. All studies with missing text, with insufficiently reported data or not written in English were also excluded.

### 3.3. Critical assessment of studies and collection of data

Data extraction was conducted in parallel by three reviewers (MFR, AD, and AI). Data regarding study details (author, year of publication), study characteristics (design, settings) and participant data (population size, incidence rate for diseases studied and the corresponding 95% CIs) were extracted.

The three reviewers independently gathered data, which they then compared and cross-checked. Missing data was sought on the journal's database and included if present. All studies with missing text or with insufficiently reported data were excluded.

All studies that reported incidence or provided sufficient information to accurately calculate the incidence of lymphomas in adult IBD patients were included. Studies focusing on pediatric populations, not reporting person-years of follow-up, or duration <1 year, and not written in English were excluded.

The following data were retrieved for each study: study design, years of follow up, surgical intervention, medical therapy, lymphoma diagnosis and incidence rate.

### 3.4. Study heterogeneity and risk of bias assessment

Assessment of heterogeneity was performed using the *I*^2^ statistic. *I*^2^ >50% was taken to represent moderate heterogeneity, and *I*^2^ >75% to represent high heterogeneity. Publication bias within studies was assessed with a funnel plot using a random effects model. Publication bias was evaluated through the Begg's and Egger's tests (16). A substantial association between the rankings of the effect estimates and the ranks of their variances is determined by Begg's test. Egger's test examines the relationship between the standardized effect estimates and the standard error using linear regression. Bias is indicated by asymmetrical Funnel plots.

### 3.5. Statistical analysis

A Freeman-Tukey transformation (17) was used to calculate the weighted summary proportion under the fixed and random effects model (18).

The correlation between immunomodulators and the risk of developing lymphoma was assessed with a relative-risk meta-analysis.

**Figure 5:**
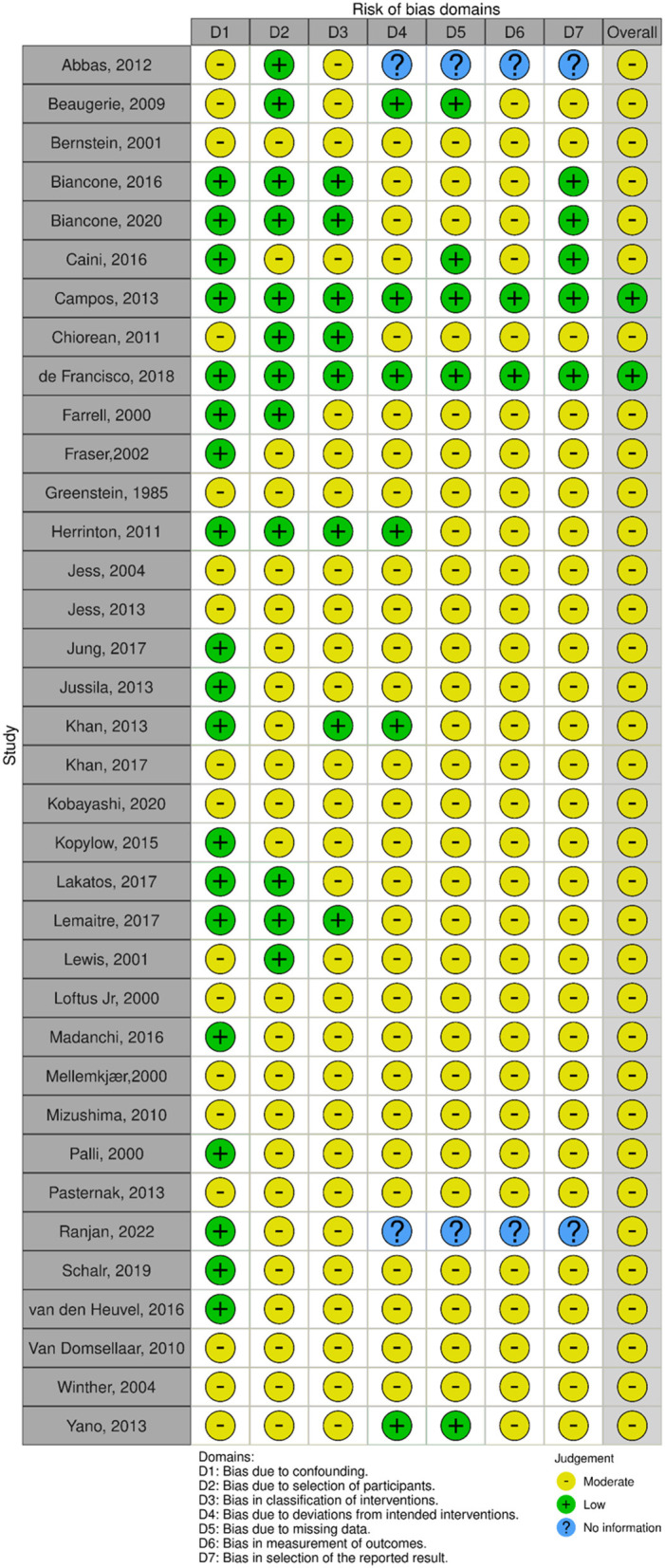
Robvis visualization tool. “Traffic light” plots of the domain-level judgments for each individual result.

Individual studies were assigned a bias risk rating using the Cochrane Collaboration's Risk of Bias Assessment Tool for Non-Randomized Studies of Intervention (ROBINS-I). The *Robvis* visualization tool was used to visualizing risk-of-bias assessments (19, 20) (Figure 5).

Individual study unadjusted incidence rates [per 100,000 person-years (py)] were calculated from the reported number of cancer cases and person-years of follow-up. Standard errors and 95% confidence intervals (CIs) were estimated assuming a Poisson distribution (21, 22). In situations with zero observed cases, the value of 3.7 was used to calculate incidence rates and the confidence interval upper limit (22).

In the inverse variance method, the weight given to each study is the inverse of the variance of the effect estimate (i.e., one over the square of its standard error). Thus, larger studies are given more weight than smaller studies, which have larger standard errors. This choice of weight minimizes the imprecision (uncertainty) of the pooled effect estimate (23–25).

Statistical analysis was performed using MedCalc Statistical Software version 19.2.6 *p* values ≤0.05 were considered statistically significant (26).

## 4. Results

**Figure 6:**
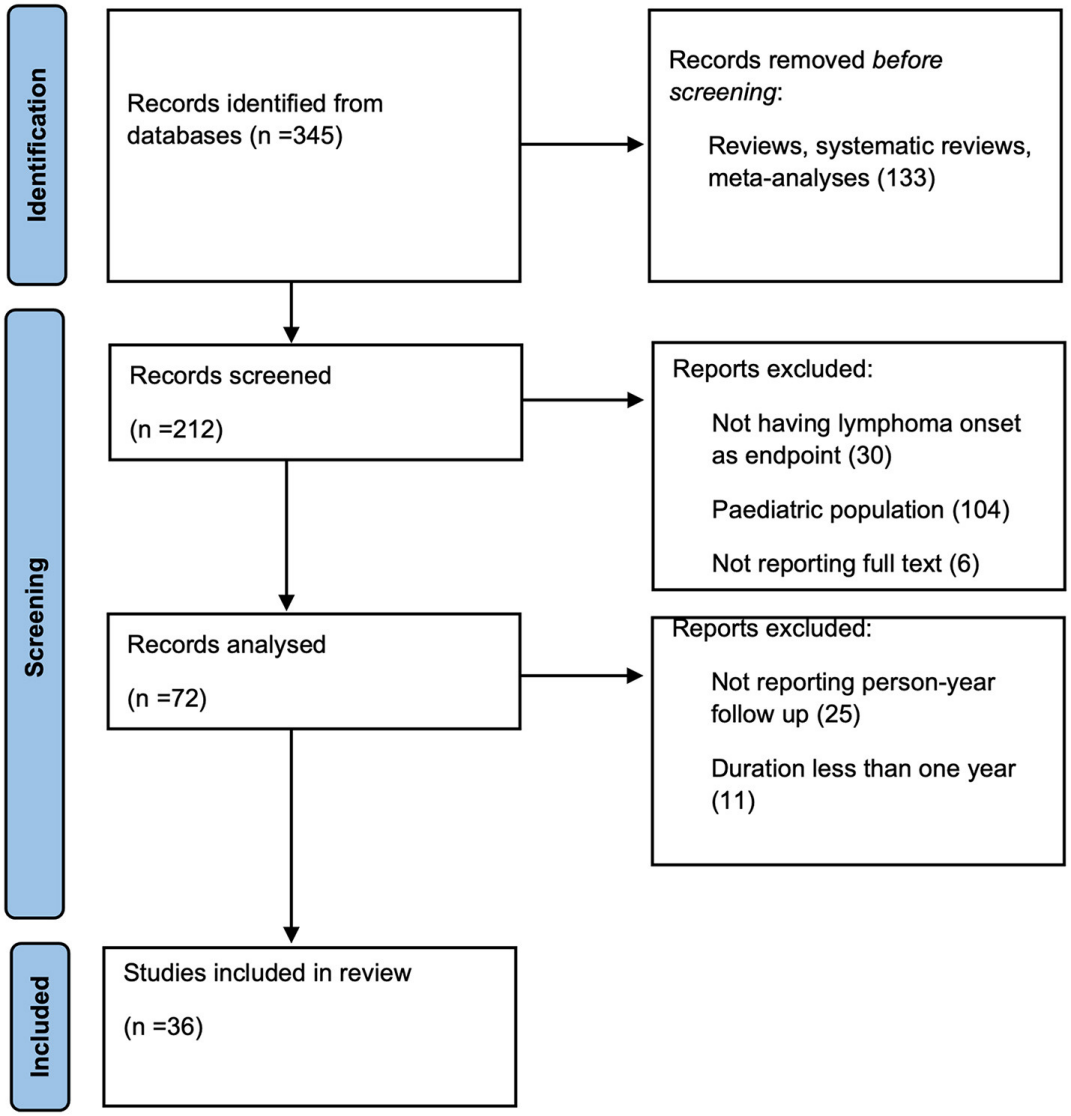
Flowchart for article selection.

A total of 345 studies were found in the electronic search. After reviewing titles and abstracts, 133 studies were excluded before screening because they were either reviews, systematic reviews or meta-analyses, while 212 were screened. One hundred forty records were excluded because lymphoma onset was not included as an endpoint, or the record did not report full text. The pediatric population was included in the study. The remaining 72 articles were analyzed; 36 articles were excluded because they did not report person-year follow-up or duration was <1 year. Eventually, a total of 36 articles comprising 6,17,386 patients were included in the final analysis (Figure 6).

Ten of the published articles were retrospective cohort studies, 8 were prospective cohort studies, 4 were case-control studies and the study design could not be determined for 14 studies. Average follow-up was an estimated 14.8 ± 12.3 years. Patients affected by CD were 1,86,074 (30.1%), while 2,78,876 (46.2%) were diagnosed with UC. The remaining 23.7% of cases were diagnosed with indeterminate colitis. Surgical intervention was necessary to treat IBD in 3,761 (0.80%) cases refractory to medical therapy.

Immunomodulators and biologic therapy were used in 24,520 (5.27%), and 17,972 (3.86%) patients, respectively (Table 1).

**Table 1:** Characteristics of included studies of lymphoma in IBD.

**References**	**Journal**	**Publication year**	**Study design**	**Person years**	**Follow-up (yrs)**	**Patients**	**Diagnosis**	**Surgery (%)**	**Immunomodulator use (%)**	**Biologic use (%)**
Abbas et al. (27)	Am J Gastroenterol	2012	Retrospective cohort	3,52,429	11.0	32,039	UC		0	0
Beaugerie et al. (6)	Lancet	2009	Prospective cohort	22,706	3.0	10,810	IBD		0	0
				10,899		5,153	CD		0	0
				11,807		5,657	UC		0	0
Bernstein et al. (28)	Cancer	2001	Case-control	41,005	14.0	5,529	IBD		0	0
				21,340		2,857	CD		0	0
				19,665		2,672	UC		0	0
Biancone et al. (29)	J Crohn's Colitis	2016	Case-control	1,32,507	3.0	44,619	IBD			
				65,859		21,953	CD	0.75		
				67,998		22,666	UC	0.07		
Biancone et al. (30)	Inflamm Bowel Dis	2020	Case-control	2,418		403	IBD			
				1,224		204	CD	59.3		
				1,134		199	UC	20.6		
Caini et al. (31)	Alimentary Tract	2016	Prospective cohort	20,509	22.3	920	IBD			
				5,128		231	CD			
				15,381		689	UC			
Campos et al. (32)	Arquivos Gastroenterol	2013	Retrospective cohort	8,442	10.5	804	CD			
				7,708	9.6	803	UC			
Chiorean et al. (33)	Dig Dis Sci	2011		30,121	8.4	3,585	IBD			
				19,127		2,277	CD			
				10,994		1,308	UC			
de Francisco et al. (34)	Alimentary Paharmacol Ther	2018	Prospective cohort	6,732	4.54	1,483	IBD	0.06	0.94	0.2
				3,941		769	CD	30.0	12.7	1.82
				2,955		651	UC	1.84	32.7	16.1
Farrell et al. (35)	Gut	2000		6,256	9.0	782	IBD		30	
Fraser et al. (36)	Aliment Pharmacol Ther	2002	Retrospective cohort	55,388	35.0	1,578	IBD		0	0
				20,494		584	CD		0	0
				34,894		994	UC		0	0
Greenstein et al. (37)	Cancer	1985	Retrospective cohort	43,142	22.0	1,961	IBD			
				26,994		1,227	CD			
				16,148		734	UC			
Herrinton et al. (9)	Am J Gastroenterol	2011		67,867	13.0	16,023	IBD		0	0
Jess et al. (38)	Aliment Pharmacol Ther	2004		6,569	35.0	374	CD			
Jess et al. (39)	Am J Gastroenterol	2013	Prospective cohort	33,843	32.0	2,211	IBD		27.2	
				11,261		774	CD		45	
				22,582		1,437	UC		18	
Jung et al. (40)	J Crohn's Colitis	2017		39,582	2.0	15,921	IBD			
				11,578		5,506	CD		60.9	0.87
				21048		9785	UC		18.8	0.54
Jussila et al. (41)	Scand J Gastroenterol	2013		2,32,526	2.0	20,970	IBD			
				51,876		4,983	CD			
				1,80,660		15,987	UC			
Khan et al. (42)	Gastroenterology	2013	Retrospective cohort	1,99,046	10.0	36,891	UC		0	0
Khan et al. (43)	Drugs Aging	2017	Retrospective cohort	1,27,518	2.0	63,759	IBD		19.3	17.0
						28,322	CD		24.1	26.5
						35,437	UC		15.5	9.45
Kobayashi et al. (44)	J Crohn's Colitis	2020		1,89,182	2.5	75,673	IBD		9.3	8.0
Kopylov et al. (45)	Inflamm Bowel Dise	2015	Case-control		5.0	19,582	IBD		8.9	0.73
Lakatos et al. (46)	J Crohn's Colitis	2012	Prospective cohort	19,293	31.0	1,420	IBD	22.8	0	0
				7,093		506	CD	41.3	0	0
				12830		914	UC	4.2	0	0
Lemaitre et al. (13)	JAMA	2017		12,68,236	6.7	1,89,289	IBD	0.18		
				6,40,097		95,537	CD		34.0	
				6,28,138		93,752	UC		19.1	
Lewis et al. (57)	Gastroenterology	2001	Retrospective Cohort	64,239	9.0	16,996	IBD		9.5	
				24,221		6,605	CD	6.7	13	
				40,018		10,391	UC	5.0	6.0	
Loftus et al. (11)	Am J Gastroenterol	2000		6,662	53.0	454	IBD			
				3,150		216	CD			
				3,512		238	UC			
Madanchi et al. (47)	Digestion	2016	Retrospective cohort	154	7.0	22	IBD			
				91		13	CD	61.5		
				63		9	UC	77.7		
Mellemkjær et al. (48)	Cancer Causes Control	2000		22,875	16.0	2,645	CD			
Mizushima et al. (49)	Digestion	2010		4,248	20.0	294	CD			
Palli et al. (50)	Gastroenterology	2000		10,592	19.0	920	IBD			
				2,716		231	CD			
				7,877		689	UC			
Pasternak et al. (4)	Am J Epidemiol	2013	Prospective cohort	3,04,992	11.0	38,772	IBD	4.0	0	0
Ranjan et al. (51)	J Gastroenterol Hepatol	2022	Retrospective Cohort	7,651	7.0	1,093	IBD			
				2,135		305	CD			
				5,516		788	UC			
Scharl et al. (52)	Am J Gastroenterol	2019	Retrospective cohort			3,119	IBD	15.1	31.2	30
						1,777	CD	40.5	33.7	12.3
						1,342	UC	10.4	28	53.5
van den Heuvel et al. (53)	IJC	2016	Prospective cohort			2,801	IBD		31.7	
				10,705	9.2	1,157	CD			
				16,193	9.8	1,644	UC			
Van Domsellaar et al. (54)	J Gastroenterol Hepatol	2010	Prospective cohort	8,563		911	IBD			
Winther et al. (55)	Clin Gastroenterol Hepatol	2004		22,290	35.0	1,160	UC			
Yano et al. (56)	J Gastroenterol Hepatol	2013		10,552	25.0	770	CD			

Overall death rate was reported by seven studies (6, 11, 27, 29–32). It was not possible to determine the incidence of lymphoma-related mortality in IBD patients due to lack of data.

### 4.1. Overall incidence of lymphomas in the IBD population

**Figure 7:**
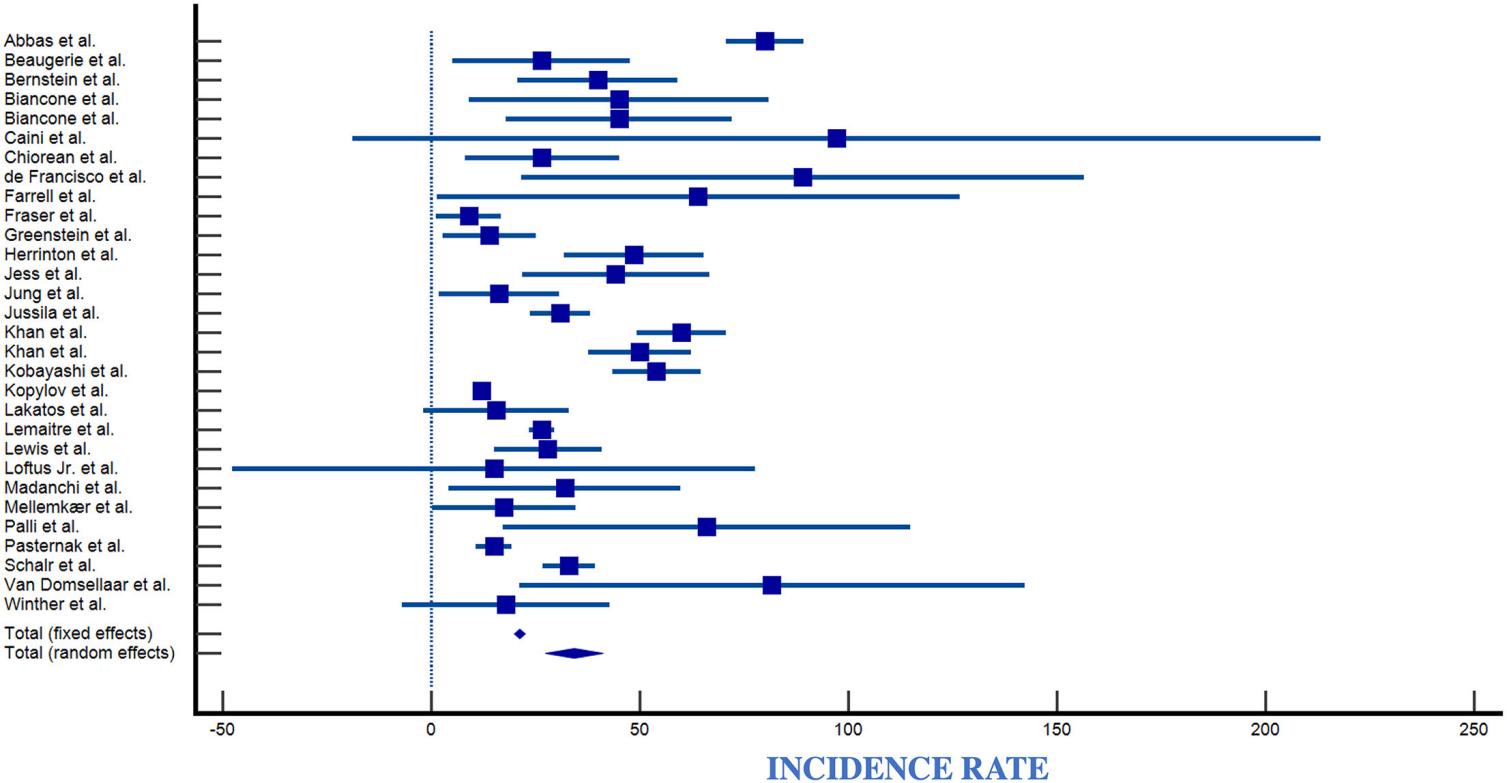
Forest plot of IBD incidence. IR, Incidence Rate; SE, Standard Error (4, 6, 9, 11, 23–27, 29–34, 36–45, 48, 50, 51, 57).

**Figure 8:**
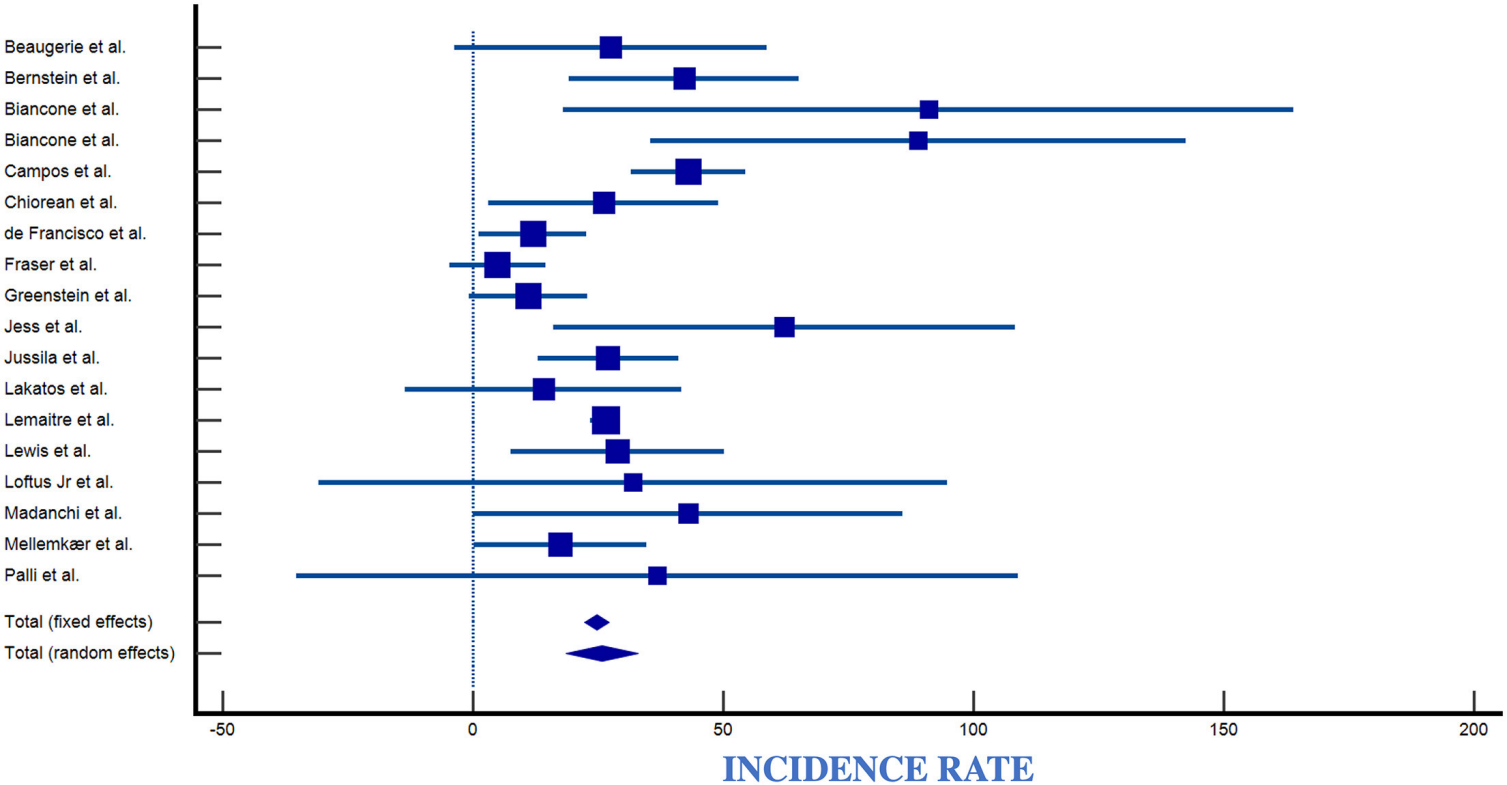
Forest plot of Crohn's disease incidence. IR, Incidence Rate; SE, Standard Error (6, 11, 24–26, 28–30, 32–34, 37, 42–46, 57).

**Figure 9:**
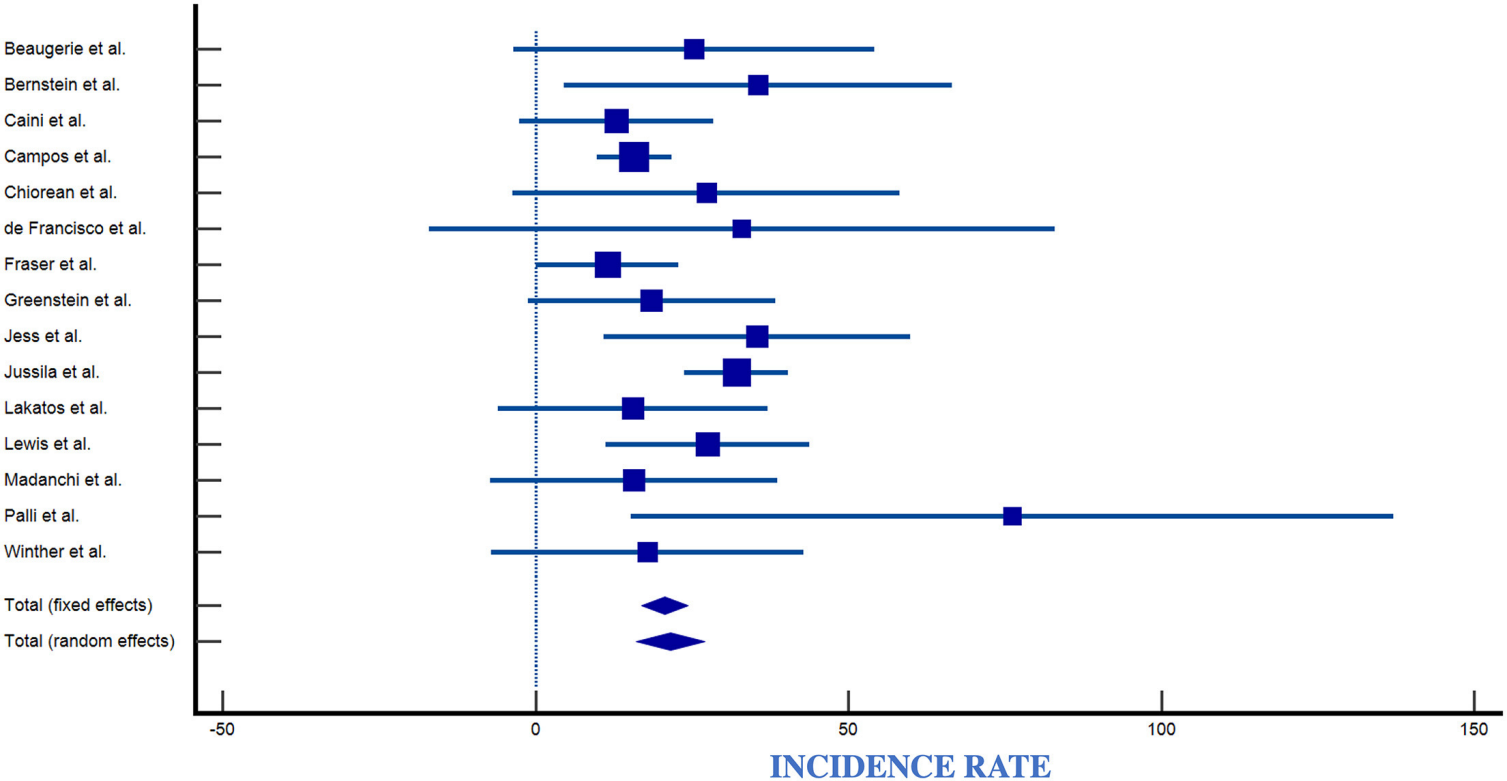
Forest plot of ulcerative colitis incidence. IR, Incidence Rate; SE, Standard Error (6, 24, 27–30, 32–34, 37, 42, 44, 45, 51, 57).

The number of observed lymphomas was 82 for CD and 507 for UC patients. Reported incidence rates for lymphoma in IBD ranged from 0.0/100,000 py (95% CI 0.0–3.7/100,000) to 89/100,000 py (95% CI 36–160/100,000) (Table 2, Figure 7). Reported incidence rates of lymphoma in CD ranged from 0.0/100,000 py (95% CI 0.0–3.7/100,000) to 91/100,000 py (95% CI 18–164/100,000) (Table 2, Figure 8). For UC, the incidence rate ranged from 0.0/100,000 py (95% CI 0.0–3.7/100,000) to 95/100,000 py (95% CI 0–226/100,000). When comparing incidence of lymphoma by sex, 389 patients were males (IR 83,66/100,000), 98 patients were females (IR 21,08/100,000), with a male-to-female ratio of ~4:1. Incidence was greatest in European countries (51%), followed by USA (17%), Asia (14.2%), Canada (8.5%), and South America (2.8%) (Table 2, Figure 9).

**Table 2:** Number of lymphomas and incidence rates in the included studies of lymphoma in IBD.

**References**	**Country**	**Male-to-female ratio**	**Diagnosis**	**Observed lymphomas (*n*)**	**Incidence rate**	**Standard error**	**95% CI lower bound**	**95% CI upper bound**
Abbas et al. (27)	Canada	N/A	UC	282	80.0	4.8	70.7	89.3
Beaugerie et al. (6)	France	3/3	IBD	6	26.4	10.8	5.3	47.5
			CD	3	27.5	15.9	−3.6	58.6
			UC	3	25.4	14.7	−3.3	54.1
Bernstein et al. (28)	Canada	N/A	IBD	16	39.9	9.8	19.9	58.1
			CD	9	42.2	11.7	14.6	69.8
			UC	7	35.6	15.8	9.2	62.0
Biancone et al. (29)	Italy	N/A	IBD	6	45	18.3	9	81
			CD	6	91	37.2	18	164
			UC	0	0.0		0.0	3.7
Biancone et al. (30)	Italy	9/2	IBD	11	45	13.77	18	72
			CD	11	89	27.3	36	143
			UC	0	0.0		0.0	3.7
Caini et al. (31)	Italy	N/A	IBD	2	97.2	59.2	0.0	232
			CD	0	0.0		0.0	3.7
			UC	2	13.0	7.9	0.0	31
Campos et al. (32)	Brasil	3/0	CD	2	43	5.86	0.0	23
			UC	1	15.8	3.06	0.0	12
Chiorean et al. (33)	USA	5/3	IBD	8	26.6	9.4	8.2	45.0
			CD	5	26.1	11.7	3.2	49.0
			UC	3	27.3	15.8	−3.6	58.2
de Francisco et al. (34)	Spain	3/3	IBD	6	89	34.4	17	160
			CD	5	12	5.48	1.5	23
			UC	1	33	25.5	0	100
Farrell et al. (35)	Ireland	3/1	IBD	4	64.0	32.0	1.3	126.7
Fraser et al. (36)	New Zealand	4/1	IBD	5	9.0	4.0	1.1	16.9
			CD	1	4.87	4.9	−4,7	14.4
			UC	4	11.5	5.8	0.2	22.8
Greenstein et al. (37)	USA	5/1	IBD	6	13.9	5.7	2.7	25
			CD	3	11.1	6.04	0.0	23.7
			UC	3	18.6	10.1	0.0	39.6
Herrinton et al. (9)	USA	28/5	IBD	33	48.6	8.5	32.0	65.2
Jess et al. (38)	Denmark	0	CD	0	0.00		0.0	3.7
Jess et al. (39)	Denmark	N/A	IBD	15	44.3	11.4	21.9	66.7
			CD	7	62.2	23.5	16.1	108.3
			UC	8	35.4	12.5	10.9	59.9
Jung et al. (40)	Korea	0/5	IBD	5	16.3	7.32	2	30.7
			CD	3				
			UC	2				
Jussila et al. (41)	Finland	41/17	IBD	72	31.0	3.7	23.8	38.2
			CD	14	27.0	7.2	12.9	41.1
			UC	58	32.1	4.2	23.8	40.4
Khan et al. (42)	USA	119/0	UC	119	60.0	5.5	49.2	70.8
Khan et al. (43)	USA	N/A	IBD	65	50	6.3	38	63
Kobayashi et al. (44)	Japan	N/A	IBD	103	54	5.38	43.9	65
Kopylov et al. (45)	Canada	0/59	IBD	121	12	1.02	10	14
Lakatos et al. (46)			IBD	3	15.5	8.9	−2.0	33.0
			CD	1	14.1	14.1	−13.5	41.7
			UC	2	15.6	11.0	−6.0	37.2
Lemaitre et al. (13)	France	192/144	IBD	336	26.5	1.53	23	29
Lewis et al. (57)	UK	7/8	IBD	18	28.0	6.6	15.1	40.9
			CD	7	28.9	10.9	7.5	50.3
			UC	11	27.5	8.3	11.2	43.8
Loftus et al. (11)	USA	N/A	IBD	1	15.0	15.0	−14.4	44.4
			CD	1	32.0	32.0	−30.7	94.7
			UC	0	0		0.0	3.7
Madanchi et al. (47)	Switzerland	N/A	IBD	5	32	14.2	4	60
			CD	4	43	21.9	0.8	87
			UC	1	15.8	11.7	0.0	46
Mellemkjær et al. (48)	Denmark	N/A	CD	4	17.5	8.8	0.4	34.7
Mizushima et al. (49)	Japan	0	CD	0	0.0		0.0	3.7
Palli et al. (50)	Italy	3/2	IBD	7	66.0	24.9	17.1	114.9
			CD	1	36.8	36.8	−35.3	108.8
			UC	6	76.2	31.1	15.2	137.2
Pasternak et al. (4)	Denmark	N/A	IBD	46	15.1	2.2	10.7	19.5
Ranjan et al. (51)	India	0	IBD	0	0.0		0.0	3.7
			CD	0			0.0	3.7
			UC	0			0.0	3.7
Scharl et al. (52)	Switzerland	N/A	IBD	103	33.02	3.26	26.6	39.4
van den Heuvel et al. (53)	Netherlands	N/A	IBD	3				
Van Domselaar et al. (54)	Spain	5/2	IBD	7	81.7	30.9	21.2	142.2
Winther et al. (55)	New Zealand	N/A	UC	2	17.9	12.7	−6.9	42.8
Yano et al. (56)	Japan	0	CD	0	0.0		0.0	3.7

### 4.2. Influence of medical therapy on lymphoma occurrence

**Figure 10:**
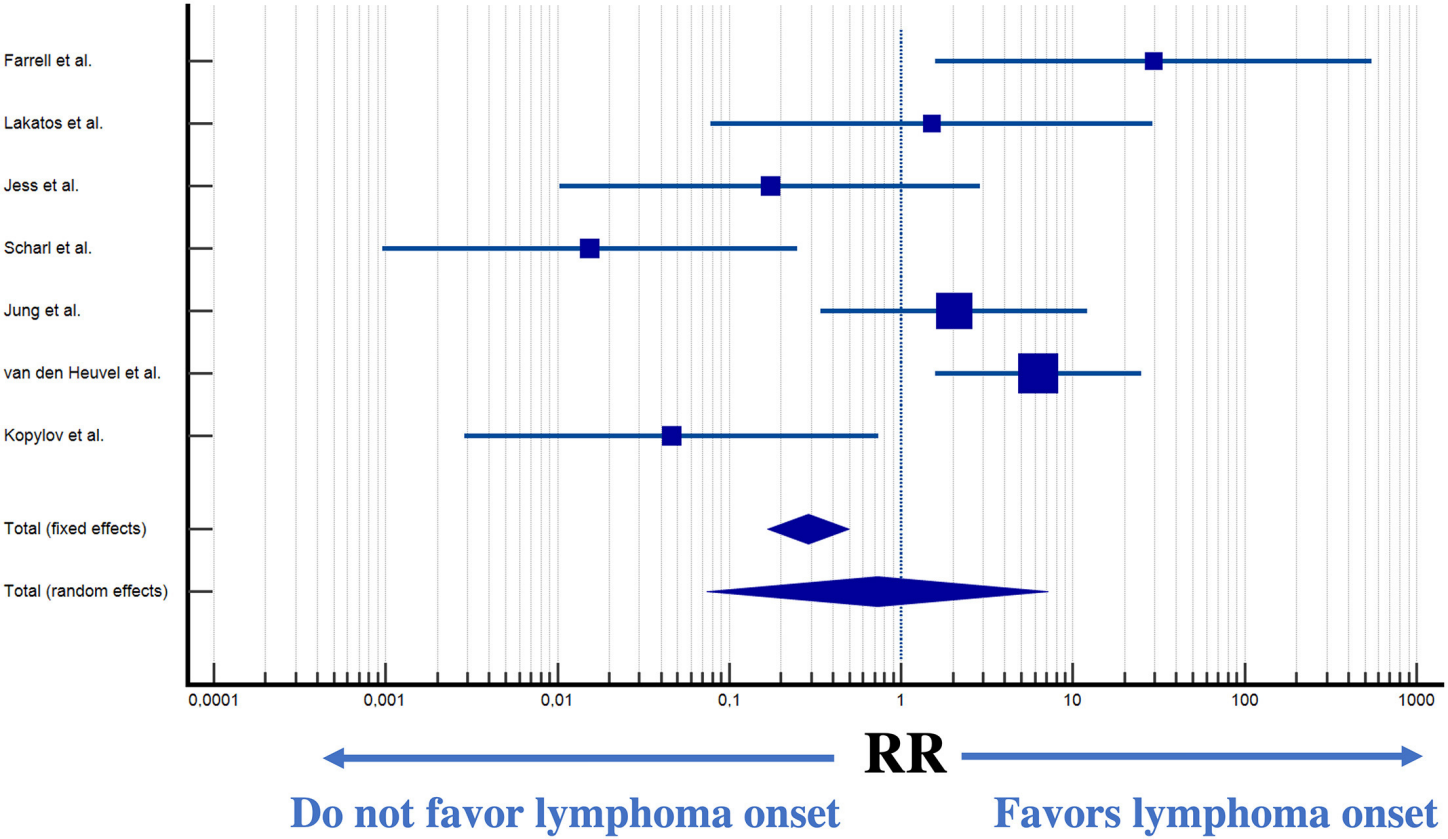
Forest plot of lymphoma incidence in patients exposed to immunomodulatory therapy (31, 34, 36, 41, 42, 48, 49).

Seven studies reported the occurrence of lymphoma in patients undergoing medical therapy. Therapy with immunomodulators was directly associated with an increased incidence of lymphoma (*p* < 0.0001) (Figure 10).

### 4.3. Heterogeneity and risk of bias assessment

Substantial heterogeneity between studies prevented the pooling of estimates (heterogeneity test, *p* < 0.001; *I*^2^ = 97.19%). Thus, the included studies are presented as unpooled estimates.

**Figure 11:**
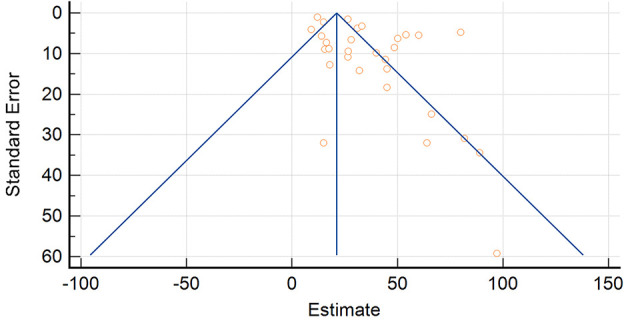
Funnel plot for inflammatory bowel disease.

**Figure 12:**
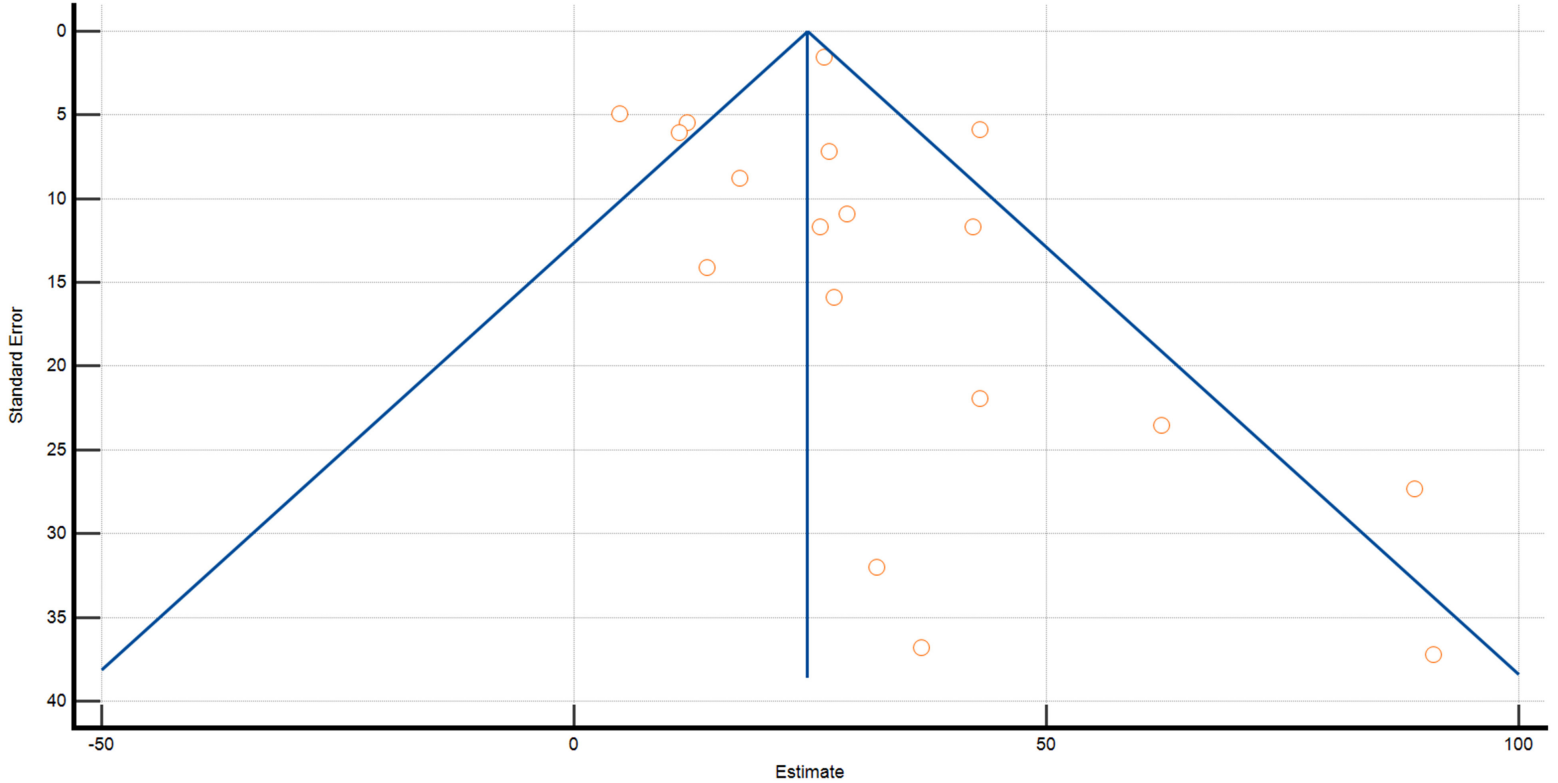
Funnel plot for Crohn's disease.

**Figure 13:**
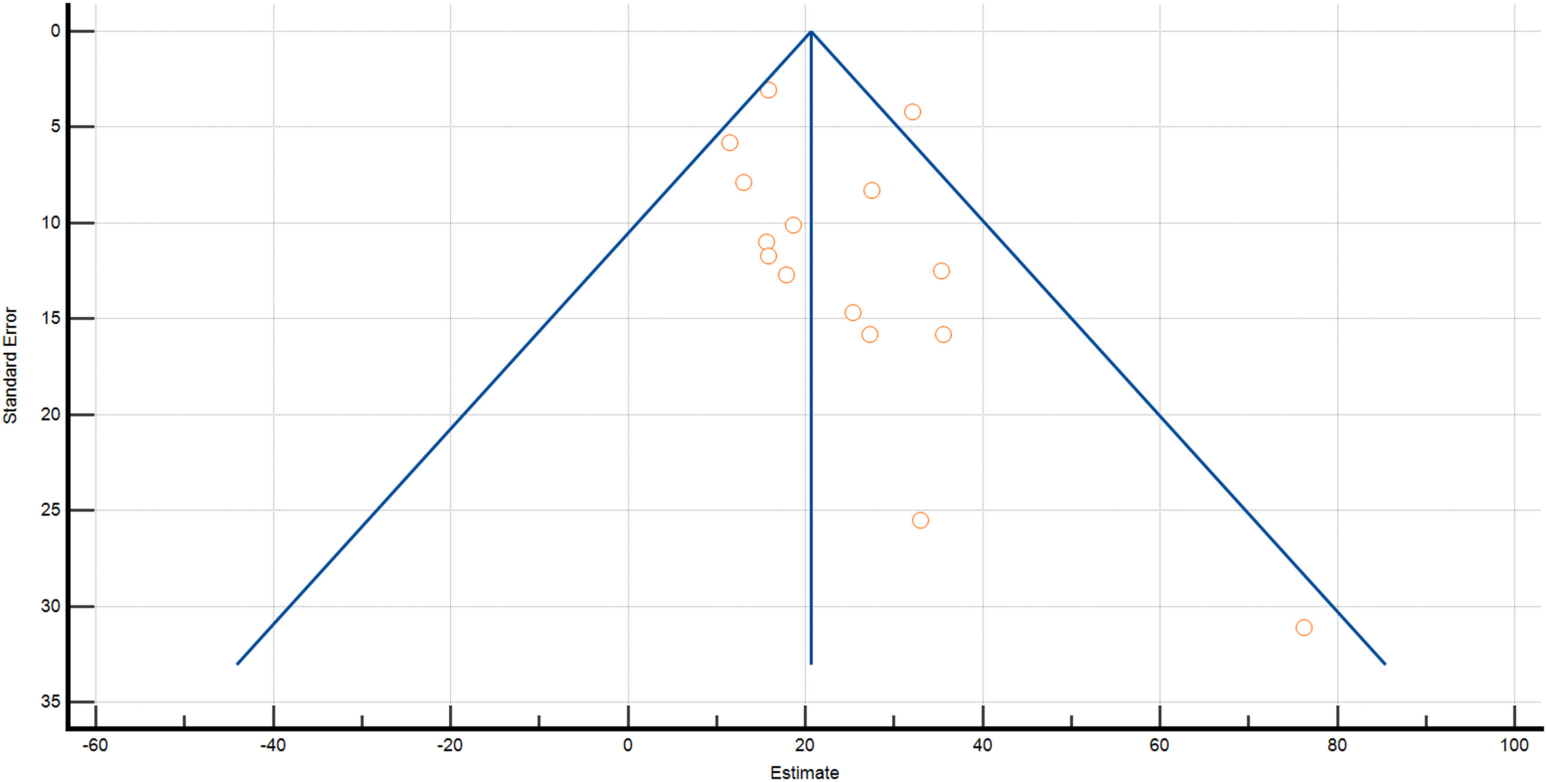
Funnel plot for ulcerative colitis.

Evidence of publication bias was overall low (*p* = 0.1941) (Figures 11–13). The *P*-values for Egger's and Begg's test for lymphoma incidence in all the IBD population was *p* = 0.0046 and *p* = 0.004603, respectively. Furthermore, *P*-value for Egger's and Begg's test in the UC subpopulation was *p* = 0.2292 and *p* = 0.0222, respectively, and *p* = 0.5326 and *p* = 0.0681 in the CD subpopulation.

## 5. Discussion

This meta-analysis was performed to produce an updated and reliable incidence rate for lymphomas in IBD patients and in CD and UC separately. Our aim was to quantify lymphoproliferative disorder incidence associated with underlying IBD. To date, the estimated worldwide Non-Hodgkin and Hodgkin lymphoma incidence is 19.0/100.000 and 2.6/100.000, respectively.^1^

In our study, the incidence was as high as 95/100.000 py for UC. Although the estimated relative risk of lymphoma in IBD may differ between studies, it has been demonstrated that there is an increased risk of lymphoma compared with the general population (5, 58–60).

This study showed a correlation between the exposure to immunomodulators and subsequent lymphoma diagnosis. Several studies demonstrated an increased risk of lymphoma in patients exposed to thiopurines (4–6, 61). The CESAME cohort included 19.486 French patients. A hazard ratio of lymphoproliferative disorders of 5.28 (95% CI, 2.01–13.9) was demonstrated in patients with a history of thiopurine treatment (6). Furthermore, in a British general practice research database including 15,471 patients, exposure to azathioprine was associated with an odds ratio of 3.22 (95% CI, 1.01–10.18) for lymphoma (62). In Western countries, an estimated 4-fold greater risk of lymphoma has been observed in patients treated with thiopurines (5, 13). However, in our study, the power of these tests was limited by incomplete reporting of variables and the small number of included studies.

The risk of lymphoma associated with anti-TNF agents remains unclear. However, being TNF, a cytokine involved in systemic inflammation and modulation of the immune system, the inhibition of TNF would be expected to favor neoplastic processes (63). Two experimental investigations back up the idea that anti-TNF, either alone or in combination with thiopurines, contributes to the development of lymphomas (64, 65). One study found that anti-TNF inhibits Natural Killer cell activity and has a detrimental effect on *in vitro* anti-lymphoma activity (64). A second, subsequent study demonstrated, in an *in vitro* lymphoma model, how Epstein-Barr virus-driven lymphomatoid transformation was amplified by both anti-TNF and 6-mercaptopurine (65).

Only two studies demonstrated an increased risk of lymphoma in IBD patients (9, 13). However, in a meta-analysis of randomized trials, monotherapy with anti-TNF agents was not associated with an increased risk of lymphoma (14). According to the prospective, 5-year, observational ENCORE registry, no excess risk of lymphoma was observed in patients diagnosed with IBD treated with anti-TNF agents alone (66). In contrast, combination treatment with thiopurines was reported to exacerbate the risk of lymphoma (67). In the present study, it was not possible to draw conclusions due to lack of information on the occurrence of lymphoma in patients treated with anti-TNF agents.

IBD alone does not seem to represent a risk factor for lymphoma development, according to the analyzed data when seen as a whole. Nevertheless, it is yet unknown whether IBD patients who have more severe and protracted disease activity are at higher risk than those who have less severe disease. Most of the body of evidence available in the literature has demonstrated that IBD patients receiving immunosuppressants such as azathioprine and mercaptopurines are at higher risk of developing lymphoma than the general population, even though this is not conclusive. Although some of these studies have taken into account potential confounders like age and sex, other potentially important factors including disease activity, duration, and co-administration of other immunosuppressive drugs have not always been included (4, 6, 9, 13, 28, 34–36, 39, 40, 42–46, 52, 57).

Limited studies have evaluated the incidence of lymphoma in IBD patients treated with azathioprine or mercaptopurines to those who were not, leading to dubious risk comparisons (14, 66, 67). Anti-TNF and thiopurine medication appears to cause a small but discernible increase in the chance of lymphoma in IBD patients, albeit this risk cannot be precisely measured (67).

Notwithstanding the fact that the largest population-based investigation to date revealed lymphoma incidence rates higher than those previously reported in several clinical trials (46), single-center experiences, and registries, the findings need to be interpreted with caution. In order to fully comprehend the complex and likely synergistic relationships between the severity and duration of IBD disease, concurrent use of immunosuppressive medications, and biologic agents, as well as how these factors relate to the development of lymphoma, well-designed Level 1 evidence needs yet to be produced.

The dysbiosis of the gut microbiota together with an imbalance of cytokines and the destruction of the mucosal barrier play a crucial role in IBD development (68). Some species of host bacteria are directly involved in the promotion of oxidative stress leading to DNA damage and subsequent cancer (69–71). The “Microbiota-gut-lymphoma axis” represents an interesting avenue of microbiota-mediated lymphomagenesis (72). The most frequent site of primary extranodal lymphoma is represented by the gastrointestinal tract and intestinal dysbiosis has been largely documented in patients with lymphoma (73). Indeed, it has been demonstrated that *H. pylori* is responsible for more than 90% of cases of gastric mucosa-associated lymphoid tissue (MALT) lymphoma (74) and that the same pattern of lesion is present in mice with chronic *Helicobacter* infection, in particular *Helicobacter Felis* (75, 76). Furthermore, bacteria such as *Campylobacter jejuni, Chlamidia psitacci* and *Borrelia bergorferi* have been involved in lymphoma development in humans (77).

Despite the findings emerging from this study, thiopurines and anti-TNF medications should be used when clinically necessary, either alone or in combination, notwithstanding the minimal absolute risk of lymphoma. While it seems higher in patients who receive combination therapy as opposed to those who have only received monotherapy, the absolute risk is still relatively small when compared to the possible complications IBD patients can develop during disease activity. Such findings might contribute to informing patients for a shared decision-making. The benefits of combination therapy prevail over the risks of malignancy in patients with severe IBD. The risk/benefit ratio could be less advantageous in individuals with mild to moderate IBD, and for these patients, monotherapy with either medication may be the best course of action.

In the clinical case presented in this study, we confirm how previous exposure to a combination of immunosuppressants and biologic agents can contribute to the subsequent development of lymphoma. The diagnosis of lymphoma, however, appeared to be difficult and delayed due to the overlapping symptoms between UC itself and lymphoma. In particular, persistent low-grade fever together with the presence of considerable abdominal lymphadenopathy found on CT-scan in a patient affected by UC and previous or current exposure to immunosuppressant and/or biologic therapy should raise suspicion of lymphoma which requires prompt histological confirmation.

The strength of the present study is the exhaustiveness of the literature search and evaluation of data for inclusion. Despite the completeness of the search, some limitations must be acknowledged. A small number of studies was included in the present review due to limited published data. In addition, substantial heterogeneity prevented pooling of estimates in some cases. Nevertheless, publication bias evaluated by both Begg's and Egger's tests was low indicating an appropriate precision of the effect estimate. Although these limitations may lead to bias in our incidence estimates, our estimates are based on the best available evidence.

In conclusion, although the risk of developing lymphomas in subjects affected by IBD is mainly linked to immunosuppressive therapy, all IBD patients should be evaluated for this possible malignant condition. When lymphoma is diagnosed, it is advised to take into account the need for modifications to IBD treatment and to take a multidisciplinary approach to both conditions. In fact, a combined multidisciplinary management and a long-term follow-up are warranted in order to decrease mortality deriving from the coexistence of both conditions.

## Data availability statement

The original contributions presented in the study are included in the article/supplementary material, further inquiries can be directed to the corresponding author.

## Author contributions

MR, LC-G, and GC contributed to the conception and design of the study. MR, AD, and AI organized the database. MR performed the statistical analysis. MR and LC-G wrote the first draft of the manuscript. LC-G and GC contributed to interpretation of results. All authors contributed to manuscript revision, read, and approved the submitted version.

## Footnotes

^1^Available online at: http://seer.cancer.gov/index.html.%20Accessed%20February%202023seer.cancer.gov/index.html (accessed February, 2023).
